# The Arclight and how to use it

**Published:** 2019-12-17

**Authors:** Obaid Kousha, Andrew Blaikie

**Affiliations:** 1 Ophthalmology Specialist Trainee, NHS Fife, Scotland, UK.; 2 Senior Lecturer, University of St Andrews, Scotland, UK.

**With hands-on training, mentorship and regular practice the latest Arclight package can be used by primary, mid-level and advanced eye care practitioners to perform comprehensive ophthalmic examinations.**

**Figure 1:**
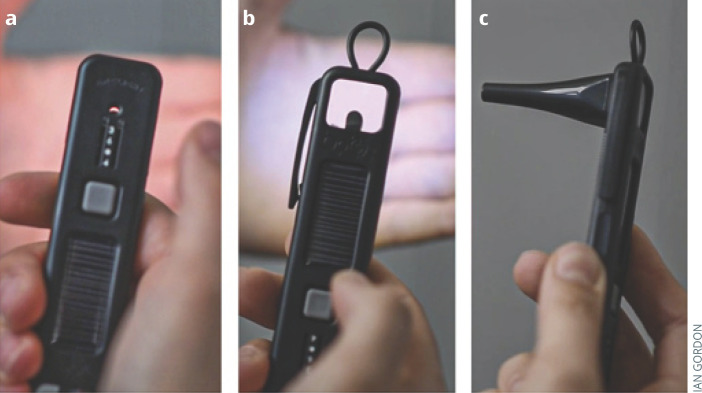
Direct ophthalmoscope (a), anterior segment loupe (b) and otoscope (c).

The Arclight is a multi-purpose medical diagnostic tool combining direct ophthalmoscopy, anterior segment loupe and otoscope (Figure 1). It was developed to overcome barriers to ownership in low-resource settings^1^ and is solar powered, uses long-lasting light-emitting diodes and costs users in low-resource regions around £10 per unit. With hands-on training (Figure 2) and ongoing mentored practice, all the major causes of treatable and preventable blindness can be reliably diagnosed.^2^,^3^ As the Arclight can also be used to examine ears^4^ and skin, the device can act as a catalyst to inter-professional education, enabling integration of eye care into universal health coverage.^1^,^5^

**Additional information**

The Arclight is available via the IAPB Standard list. Visit **https://iapb.standardlist.org/the-products/arclight-mk3-5-ophthalmoscope-otoscope**

The manufacturer's website is **www.arclightscope.com**

For training videos and information, visit **http://med.st-andrews.ac.uk/arclight/training/**

**Figure 2:**
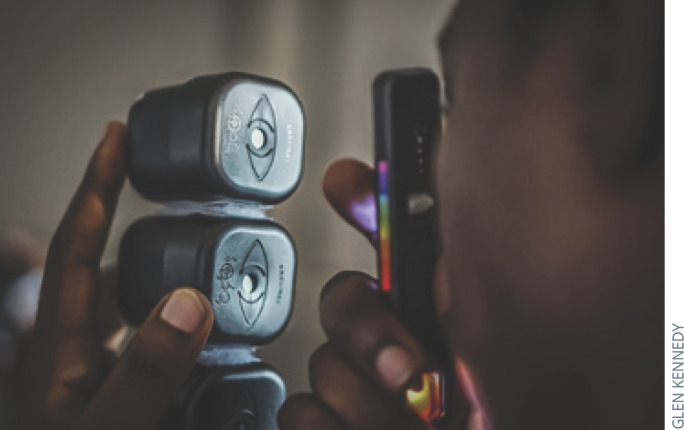
Hands-on training with simulation tools

## Anterior segment examination

**Figure 3:**
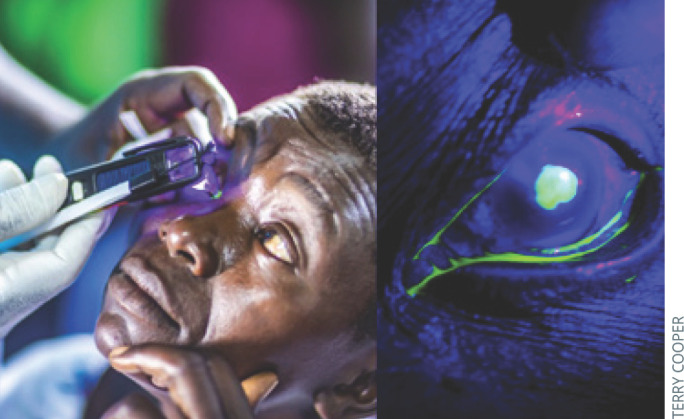
Anterior segment loupe: blue light & fluorescein to highlight ulcers

Using the internally lit 14 dioptre lens, the lids, conjunctiva, cornea and anterior chamber can be examined. The blue light highlights fluorescein staining, which enables epithelial loss and the activity of ulcers to be seen clearly (Figure 3). The headband allows you to conduct a hands-free examination, which simplifies the removal of foreign bodies and aids trachoma tarsal plate examination.^6^ Precise differentiation of corneal scarring from cataract can also be achieved, avoiding needless referrals to distant cataract surgery centres.

## The red reflex

**Figure 4:**
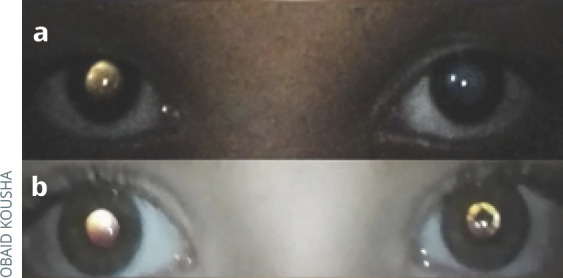
a) Right eye: normal reflex. Left eye: no reflex due to complete cataract b) Right eye: normal reflex. Left eye: posterior polar cataract

In children with darker skin and a pigmented fundus, the so-called ‘red’ reflex appears paler. The reflex can be observed using the direct ophthalmoscope (Figure 4). Media opacity, due to cataract or retinoblastoma, can be reliably screened for in babies as well as adults.^7^ Hold the device at arm's length and illuminate both eyes at the same time (select the brightest light). The examination is best performed in a dimly lit room or, even better, under a black-out cloth. With experience, squint and refractive error can also be identified,^8^ which can improve the quality of referral to paediatric services.

## Direct ophthalmoscopy

**Figure 5:**
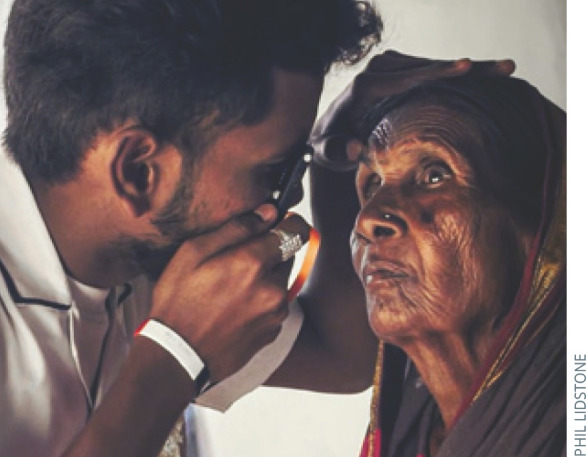
Direct ophthalmoscopy: approach from 15 degrees temporal on the horizontal plane to see the optic nerve

After placing your feet next to the patient in the position you aim to finish, lean back and follow the red reflex in towards the patient's eye on the horizontal plane at 15 degrees temporal (Figure 5). Use the right hand and right eye to examine the patient's right eye and vice versa. This ‘flight path’ should bring the disc into view. If the disc is not seen, follow the ‘arrows’ created by the branches of retinal vessels as they point towards the disc.

**Figure 6:**
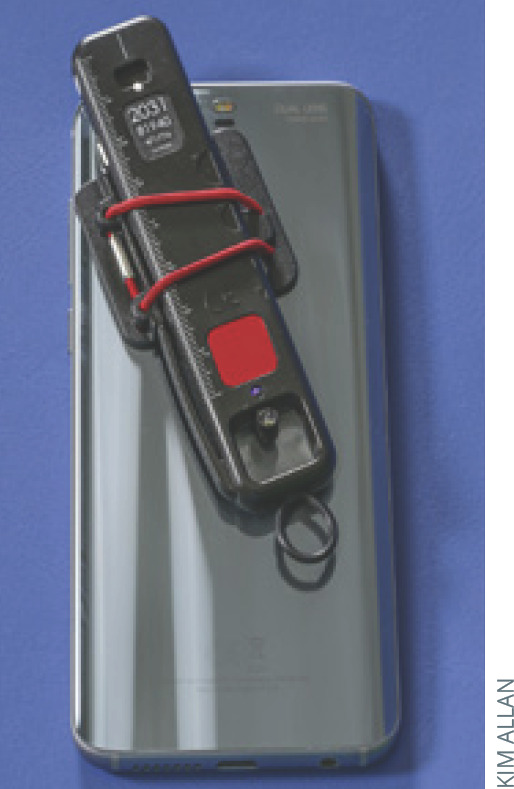
Optic nerve examination and imaging with mobile phone clip

Assessing the margin, the colour of the neuro-retinal rim and the cup to disc ratio can help to identify raised intraocular pressure, glaucoma and optic atrophy (Figure 6). After examining the four major retinal vessel branches and the surrounding retina, ask the patient to look at the light. This will bring the central macula (fovea) into view. Macular disease due to infection, diabetes and ageing can now be seen.

## Binocular indirect ophthalmoscopy

Quick and simple binocular indirect ophthalmoscopy can be performed on well dilated patients. By placing the light source of the direct ophthalmoscope directly between the user's eyes, a wide field view of the fundus can be achieved with the addition of a standard condensing lens.

## Mobile phone camera clip

Where diagnostic uncertainty occurs, you can take video by attaching the ophthalmoscope, loupe or otoscope to a mobile phone camera (Figure 6) using a universal clip.^9^ This offers the opportunity for a remote second opinion or for documenting clinical signs for later comparison.

## Other tools

To complete a comprehensive ophthalmic examination, the Arclight package comes with several other tools, including a distance/near visual acuity chart and matching card, an engaging ‘bird’ near target and flashing white-blue ‘lure’ for children, a red desaturation square and a white target for visual field assessment as well as a ruler, pupilometer and cup to disc ratio gauge.
